# CO_2_ fractional laser-assisted transdermal delivery of silk nanofiber carriers in a rabbit ear hypertrophic scar model

**DOI:** 10.1093/burnst/tkac040

**Published:** 2022-11-11

**Authors:** Yan Yang, Lutong Liu, Xiaojin Wu, Xue Wang, Qiang Lu, Zhen Zhang

**Affiliations:** Department of Dermatology, Shanghai Ninth People's Hospital, Shanghai Jiao Tong University School of Medicine, Shanghai 200011, P. R. China; National Engineering Laboratory for Modern Silk and Collaborative Innovation Center of Suzhou Nano Science and Technology, Soochow University, Suzhou 215213, P. R. China; Department of Dermatology, Shanghai Ninth People's Hospital, Shanghai Jiao Tong University School of Medicine, Shanghai 200011, P. R. China; Department of Dermatology, Shanghai Ninth People's Hospital, Shanghai Jiao Tong University School of Medicine, Shanghai 200011, P. R. China; National Engineering Laboratory for Modern Silk and Collaborative Innovation Center of Suzhou Nano Science and Technology, Soochow University, Suzhou 215213, P. R. China; Department of Dermatology, Shanghai Ninth People's Hospital, Shanghai Jiao Tong University School of Medicine, Shanghai 200011, P. R. China

**Keywords:** CO2 fractional laser, Hypertrophic scar, Silk nanofiber hydrogels, Transdermal delivery, Laser-assisted drug delivery

## Abstract

**Background:**

Hypertrophic scars are skin fibrotic diseases, characterized by fibroblast hyperproliferation and excessive accumulation of extracellular matrix. However, topical drug application for hypertrophic scars are unsatisfactory. The purpose of this study was to explore the permeability of silk nanofiber hydrogels (SNFs) loaded with rhodamine 6G (R6G) and rhodamine 110 (R110) mediated by CO_2_ fractional laser irradiation into hypertrophic scar tissues.

**Methods:**

In this work, R6G and R110 were chosen as hydrophilic and hydrophobic model molecules. They were loaded inside SNFs. *In vivo* rabbit ear hypertrophic scars were treated with CO_2_ fractional laser irradiation and then R6G/R110-laden SNFs were applied to the scars to evaluate their synergetic effect on drug penetration efficiency. Their permeability was quantified by fluorescence intensity and measured by confocal laser scanning microscopy on days 1, 3, 5 and 7. More specifically, the thermal coagulation zone (CZ) and its surrounding area (peri-CZ) caused by the thermal coagulation of the laser were discussed separately.

**Results:**

Our data indicated that the SNFs promoted the penetration of R6G but not that of R110 in the peri-CZ on day 1 when combined with laser irradiation. Interestingly, both R6G and R110 were abundant in the CZ and remained stable on days 1, 3 and 5. Moreover, rapid re-epithelialization hindered the long-term permeability of both drugs.

**Conclusion:**

Combining CO_2_ fractional laser irradiation with SNF drug delivery could improve the efficiency of hydrophilic drug delivery within 24 h before total re-epithelialization.

Highlights

- Silk nanofiber hydrogels provide a suitable platform for revealing the permeability of different drugs into skin treated with CO_2_ fractional laser irradiation due to their good loading capacity for both hydrophilic and hydrophobic drugs.
- CO_2_ fractional laser irradiation was shown to enhance the permeability of hydrophilic and hydrophobic drugs in rabbit ear hypertrophic scars.
- Silk nanofiber hydrogels combined with CO_2_ fractional laser irradiation could improve the skin penetration of hydrophilic drugs within 24 h after laser treatment (before total re-epithelialization).

## Background

Abnormal wound healing usually results in hypertrophic scars (HSs) or even keloids [1,2]. HSs cause organ dysfunction and motor incapacity, imposing a substantial financial burden on patients worldwide [3,4]. HS treatment includes excision, radiation therapy and the intralesional injection of steroids, 5-fluorouracil or botulinum toxin A [5]. However, researchers are still searching for more effective and convenient HS treatment [6,7]. Surgical incisions are invasive procedures that are likely to result in new scars [8]. Intralesional injections are often accompanied by adverse effects and fail to maintain a long-lasting high local concentration at the injection site [9,10]. Topical drug application has the advantages of high specificity, convenience and few side effects but is less effective [11]. Compared to normal skin, HS has a thicker dermis with denser collagen fibers [12]. Therefore, enhancing transdermal efficiency is a key factor in the treatment of HS.

Based on dot-matrix photothermolysis theory, ablative fractional lasers (AFLs) have been proven to be able to increase the dermal absorption of topically administered drugs [13,14] by disrupting the stratum corneum barrier temporarily [15,16]. AFLs have been extensively used to increase clinical efficacy in the treatment of skin diseases such as nonmelanoma skin cancer, vitiligo, melasma, scarring and alopecia [17–20]. As an AFL, a CO_2_ fractional laser has been confirmed to improve the elasticity, thickness, appearance and symptoms of mature hypertrophic burn scars [21]. However, most research currently focuses on *in vitro* experiments without long-term observation. Therefore, dynamic drug distribution over time has not been observed [22–24]. Additionally, due to rapid re-epithelialization [25], short drug penetration time intervals limit the use of CO_2_ fractional laser-assisted drug delivery [15,26]. As such, the application of CO_2_ fractional laser-assisted drug delivery for the treatment of scars deserves more research attention with regard to enhancing the penetration of drugs.

In recent years, multiple nanocarriers with transdermal capacities have been developed and applied in AFL-assisted drug delivery, improving the therapeutic effect of drugs on HSs [27]. Silk nanofiber hydrogel (SNF) is a type of natural nanofiber with a high biocompatibility, good water dispersibility, tailorable biodegradability, low bacterial attachment and good mechanical characteristics [28]. The SNF structure is characterized by an abundance of β-sheet structures, which enhance its mechanical properties. Moreover, SNFs contain various functional groups, such as amines, phenol and alcohol, which make it possible to incorporate various drugs within or on the surface of SNFs. In addition, SNFs enhance drug concentrations in lesions as drugs are delivered at a sustained rate [29,30]. Thus, SNFs have been introduced as powerful drug carriers that can be applied in different biomedical fields [31,32]. It is anticipated that drug-laden SNFs cover the HSs treated by CO_2_ fractional lasers to achieve better transdermal delivery.

In this study, we aimed to reveal whether CO_2_ fractional laser-mediated drug-laden SNFs could penetrate HSs more effectively. Water-soluble rhodamine 6G (R6G) and water-insoluble rhodamine 110 (R110) as hydrophilic and hydrophobic drug models were loaded on SNFs. When treatment with a CO_2_ fractional laser was applied in a rabbit HS model, the drug-laden SNFs were applied to the HSs to evaluate transdermal penetration *in vivo*. Additionally, the week-long *in vivo* experiment provided a link between the healing of laser channels and drug penetration for future studies.

## Methods

### Preparation of SNFs

The original silk fibroin solution (6 wt%) was prepared through previously reported procedures [32]. It was concentrated to ~20 wt% over 24 h at 60°C to form metastable nanoparticles and then diluted to 2 wt% with deionized water. This diluted silk solution was incubated at 60°C in a constant-temperature drying oven for >24 h until hydrogels formed. The SNF preparation was conducted by the National Engineering Laboratory for Modern Silk & Collaborative Innovation Center of Suzhou Nano Science and Technology (Soochow University, Suzhou 215 123, People’s Republic of China).

**Figure 1.**
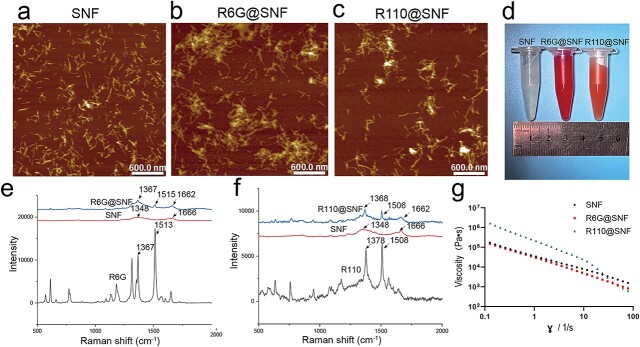
Characterization of SNF, R6G@SNF and R110@SNF. (**a**, **b**, **c**) AFM images of SNF, R6G@SNF and R110@SNF. Scale bar: 600 nm. (**d**) Images of SNF, R6G@SNF and R110@SNF. (**e**, **f**) Raman spectra and (**g**) viscosity of SNF, R6G@SNF and R110@SNF. The silk concentration was 2 wt% for all hydrogels. *SNF* silk nanofiber hydrogel, *R6G* rhodamine 6G, *R110* rhodamine 110, *R6G@SNF* 0.03 wt% R6G-laden silk nanofiber hydrogel, *R110@SNF* 0.03 wt% R110-laden silk nanofiber hydrogel, *AFM* atomic force microscopy

### Preparation of 0.03% R6G@SNF and 0.03% R110@SNF

Hydrophobic and hydrophilic drugs were loaded on SNFs via various processes. For hydrophilic R6G (Acros Organics), the drug was added to the SNF solution directly and then loaded on the nanofibers after stirring for 4 h at room temperature. Hydrophobic drugs were loaded on the SNF through a blending–centrifuging process. R110 (Shanghai Yien Chemical Technology Co. Ltd) was dissolved in ethanol and then blended with SNF aqueous solution. The blend solution was stirred for 24 h at room temperature to induce the transfer of R110 from ethanol to the SNF. Then, the blend solution was centrifuged at 10000 rpm for 20 min to separate the R110-laden SNF from ethanol. The specific steps were as follows. R6G (3 mg) was dissolved in 100 μl of deionized water and the solution was added to 10 ml of 2 wt% SNF, immediately followed by 4 h of stirring using magnetic stir bars at room temperature. R110 (18 mg) was dissolved in 1 ml of 100% ethanol and mixed completely. The solution was slowly added to 20 ml of 2 wt% SNF and swirled for 24 h at room temperature. Then, the mixture was transferred to a centrifuge tube and subsequently centrifuged at 10000 rcf for 20 min to obtain molecule-loaded nanofibers. The supernatant was collected after washing with deionized water three times for the quantitative analysis of the unloaded R110 content using a UV–vis spectrophotometer (Cary5000, Agilent, Santa Clara, USA). Through optimizing the conditions, the R6G and R110 loading capacities were 0.03 and 0.18%, respectively. R110@SNF (0.18%) was diluted with 2 wt% SNF to eventually obtain 0.03% R110@SNF. For the control, we also prepared a 0.03% R6G aqueous solution and a 0.03% R110 ethanol solution. The whole process was shielded from light.

### Characterization

The sample morphology was examined by atomic force microscopy (AFM, Bruker Dimension ICON, Germany). Samples were diluted to 1:1000 and dispersed in ultrasound for 10 min. Then, a 2 μl solution was spin-coated onto a clean mica surface and the surface was blown with nitrogen. A confocal Raman spectrometer (InVia Qontor, Renishaw, 785 nm diode laser) was used to evaluate the loading of R6G and R110 in the SNFs. The rheological properties of all hydrogels were measured with a rheometer (Mars40, Thermo Fisher Scientific, USA). Frequency sweeps were collected continuously over a wide frequency range from 0.1 to 100 rad s^−1^ at 25°C.

**Figure 2.**
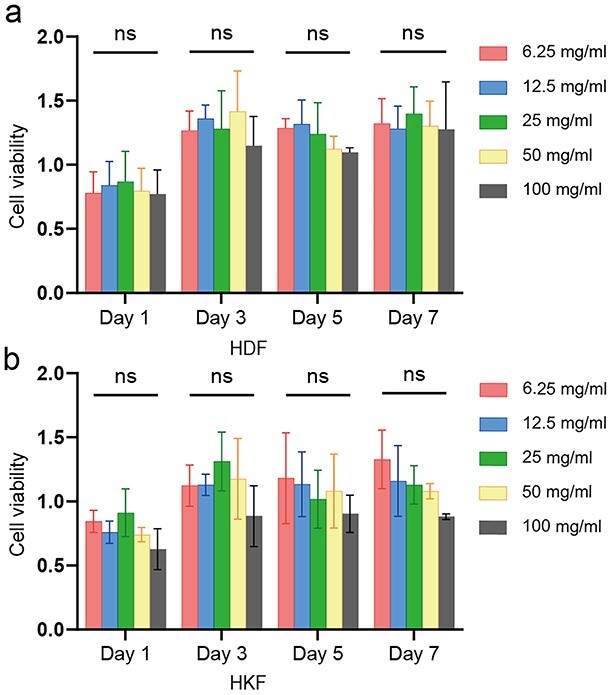
Cytocompatibility and cytotoxicity of SNF. (**a**) Cell viability in HDF. (**b**) Cell viability in HKF. SNF concentrations were 6.25, 12.5, 25, 50 and 100 mg/ml, respectively. Detection time points were 1, 3, 5 and 7 days after SNF application. Statistical comparisons were made using two-way ANOVA with Tukey’s multiple comparisons; ns, no statistical significance. Data presented as the mean ± standard deviation, *n* = 3. *SNF* silk nanofiber hydrogel, *HDF* human dermal fibroblasts, *HKF* human keloid fibroblasts, *ANOVA* analysis of variance

**Figure 3.**
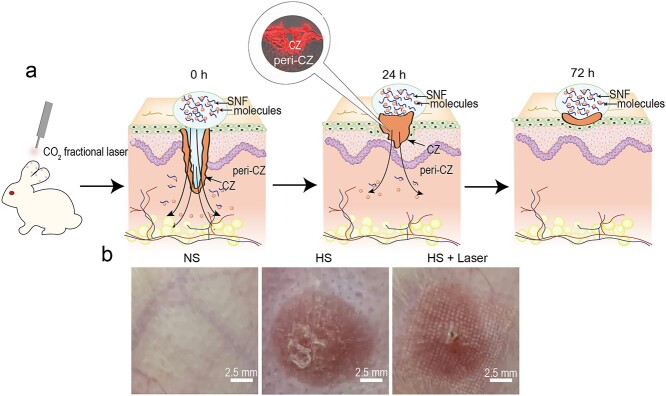
CO_2_ fractional laser-assisted transdermal delivery of SNF in a rabbit ear HS model. (**a**) Schematic diagram of transdermal delivery of drug-laden SNF in laser channels at 0, 24 and 72 h. (**b**) Photographic images of the normal skin (NS), HS and HS + laser on rabbit ears. Scale bar: 2.5 mm. *SNF* Silk nanofiber hydrogel, *NS* normal skin, *HS* hypertrophic scar, *HS + laser* hypertrophic scar with laser treatment

**Figure 4.**
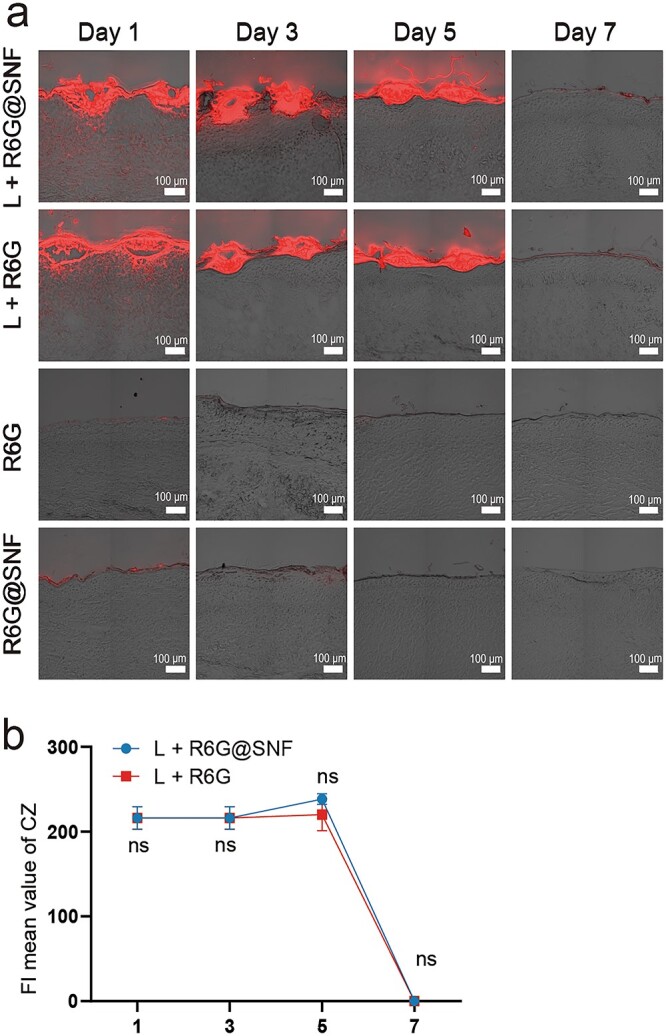
Penetration of R6G@SNF and R6G in rabbit ear HSs *in vivo*. (**a**) Confocal fluorescence images of cross-sections of rabbit HSs with different treatments. L + R6G@SNF: laser treatment and R6G@SNF application; L + R6G: laser treatment and R6G application; R6G: R6G application only; R6G@SNF: R6G@SNF application only. Scale bar: 100 μm. (**b**) Fluorescence intensity (FI) mean value of CZ. Statistical comparisons were made using two-way ANOVA with Tukey’s multiple comparisons; ns, no statistical significance. Data presented as the means ± standard deviation, *n* = 6. *R6G* Rhodamine 6G, *R6G@SNF* 0.03 wt% R6G-laden silk nanofiber hydrogel, *HSs* hypertrophic scars, *L* laser, *CZ* coagulation zone, *ANOVA* analysis of variance

### Isolation and culture of human keloid fibroblasts and human dermal fibroblasts

Human keloid tissues were obtained from 18- to 45-year-old Chinese plastic surgery patients with informed consent at Shanghai Ninth People’s Hospital. To collect human dermal fibroblasts (HDFs), foreskins were obtained from Chinese circumcision patients with informed consent at Shanghai Ninth People’s Hospital. This study was performed according to the ethical guidelines of the 1975 Declaration of Helsinki and approved by Shanghai Ninth People’s Hospital. The human tissues were rinsed in Dulbecco’s phosphate buffered saline, the epidermis tissue was cut into small pieces (1–2 cm^3^), and subcutaneous fat was removed and subsequently soaked in cell culture medium (Gibco) containing 2% penicillin–streptomycin (Gibco) and 10% foetal bovine serum (Gibco) for 30 min. Next, the small tissues were minced and digested in cell culture medium containing 0.2% collagenase (Collagenase NB 4 Standard Grade, Nordmark, Germany) for 2–4 h in a 37°C incubator. Afterwards, the cell suspension was filtered through a 70 μm filter and centrifuged at 1200 rpm for 5 min. Finally, the cell pellet was resuspended in cell culture medium (Gibco) supplemented with 10% foetal bovine serum (Gibco) and 1% penicillin–streptomycin (Gibco), and the cells were cultured at 37°C in 5% CO_2_ with the medium changed every 3 days. For experiments, the cell passage was ≤5.

### *In vitro* cytocompatibility of SNFs

Cell proliferation was evaluated with a cell counting kit 8 (CCK-8, Dojindo Laboratories, Kumamoto, Japan). Human keloid fibroblasts (HKF) and HDF cells were seeded in 96-well plates with 2000 cells per well. After 24 h of incubation, they were treated with 0.5, 1, 2, 4 or 8 mg/ml SNF dissolved in cell culture medium for 1, 3, 5 or 7 days. At the corresponding time, cell culture medium containing 10% CCK-8 solution was added to each well and incubated for 1 h at 37°C. Finally, the absorbances were obtained at 450 nm using a microplate reader (Multiskan FC, Thermo Scientific).

### Rabbit ear HS model

Two-week-old male New Zealand white rabbits weighing 2 kg (SLAC, Shanghai) were kept individually. Xylazine Hydrochloride was combined with 1.5 mg ketamine per 100 g weight of rabbits and administered intramuscularly. All of the wounds were located around the center point of the rabbit ears and were distributed equally. Each circular wound was removed with a diameter of 1 cm of full-thickness skin and perichondrium while the cartilage remained intact. The rabbit ear wounds healed and were repaired entirely, with a noticeable diameter of ~0.9 cm of HSs after 28 days. The scars appeared as bright red bumps on the skin, with obvious hyperplasia and characteristics of being thick and hard.

### *In vivo* transdermal drug penetration assay in rabbit ear HS model

Mature rabbits with hypertrophic ear scars were randomly divided into eight groups: the control groups received topical treatment with R6G, R6G@SNF, R110 or R110@SNF, and the laser irradiation groups additionally received topical CO_2_ fractional laser treatment (L + R6G, L + R6G@SNF, L + R110 or L + R110@SNF). The laser was operated in DeepFX mode under the parameters of 25 MJ energy intensity, 20% coverage, 300 Hz emission frequency and on the 10th spot size without overlap. The rabbits were well anesthetized before laser irradiation via intramuscular injection anesthesia. All groups were subsequently given the corresponding drug treatments (50 μL drug/HS). After 1, 3, 5 and 7 days of treatment, rabbits were sacrificed by intravenous injection of an overdose of sodium pentobarbital and HS tissues were excised. These HS tissues were then cut into frozen sections with a 7 μm thickness. Finally, the frozen sections were viewed under a Zeiss LSM 880 confocal microscope (Zeiss Co., Germany). The fluorescence intensity was measured by ZEN 2.6 software.

**Figure 5.**
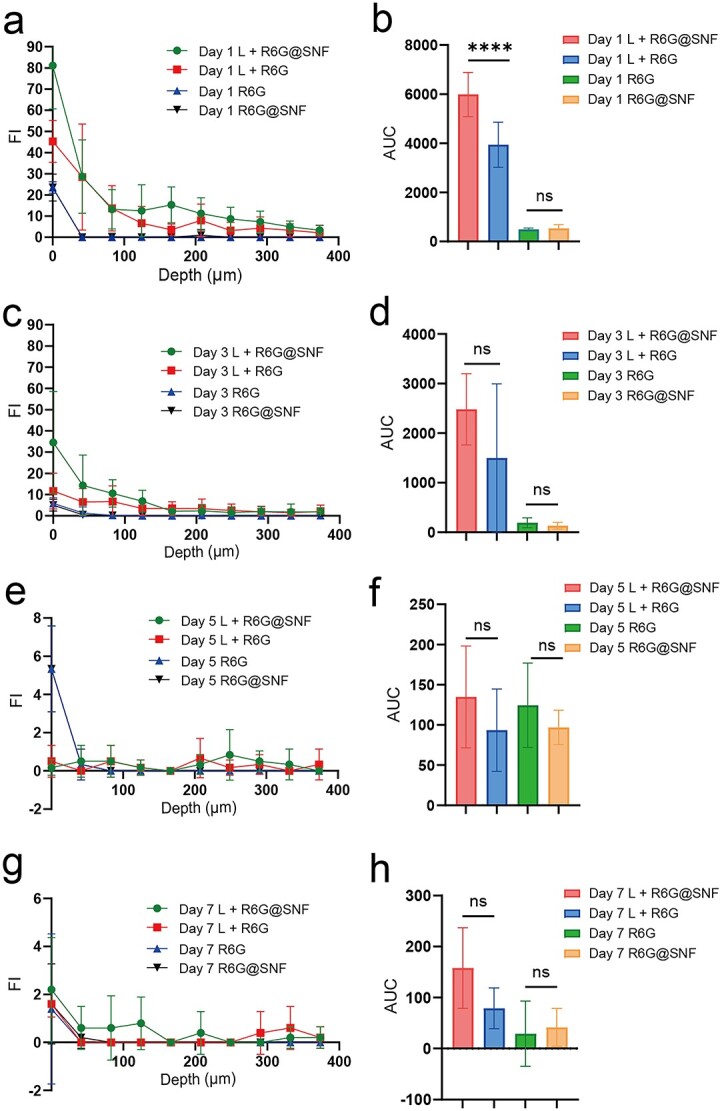
Comparison of R6G@SNF and R6G penetration in rabbit ear HSs *in vivo*. (**a**, **c**, **e**, **g**) Fluorescence intensity (FI) curves with penetration depth. (**b**, **d**, **f**, **h**) AUC of each group. Statistical comparisons were made using one-way ANOVA with Tukey’s multiple comparisons; ns, no statistical significance, ^*^^*^^*^^*^p ≤ 0.0001. Data presented as the means ± standard deviation, *n* = 6. *R6G* Rhodamine 6G, *R6G@SNF* 0.03 wt% R6G-laden silk nanofiber hydrogel, *HSs* hypertrophic scars, *AUC* area under the fluorescence intensity curves, *ANOVA* analysis of variance

**Figure 6.**
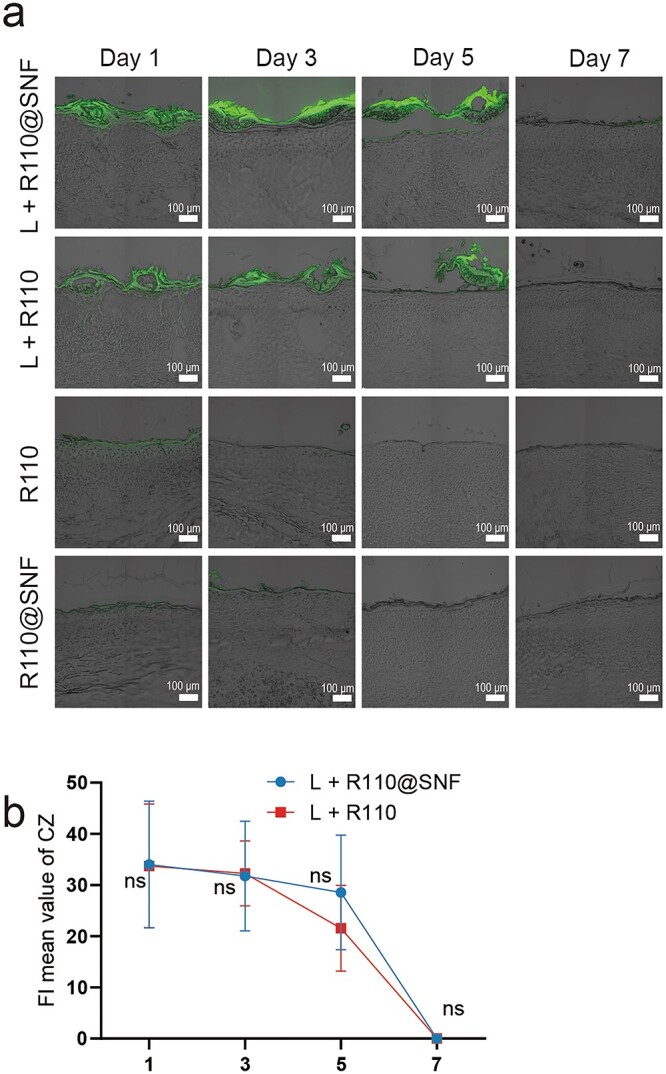
Penetration of R110@SNF and R110 in rabbit ear HSs *in vivo*. (**a**) Confocal fluorescence images of cross-sections of rabbit HSs with different treatments. L + R110@SNF: laser treatment and R110@SNF application; L + R110: laser treatment and R110 application; R110: R110 application only; R110@SNF: R110@SNF application only. Scale bar: 100 μm. (**b**) Fluorescence intensity (FI) mean value of CZ. Statistical comparisons were made using two-way ANOVA with Tukey’s multiple comparisons; ns, no statistical significance. Data presented as the means ± standard deviation, *n* = 6. *R110* rhodamine 110, *R110@SNF* 0.03 wt% R110-laden silk nanofiber hydrogel, *HSs* hypertrophic scars, *L* laser, *CZ* coagulation zone, *ANOVA* analysis of variance

**Figure 7.**
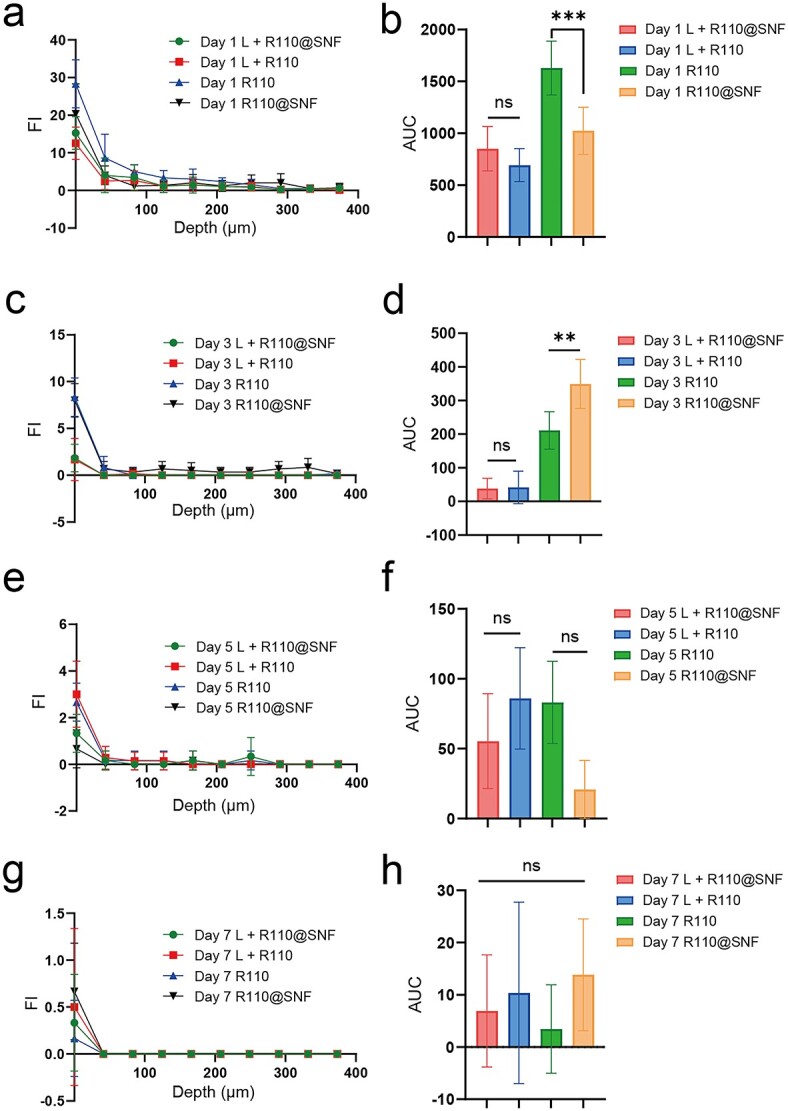
Comparison of R110@SNF and R110 penetration in rabbit ear HSs *in vivo*. (**a**, **c**, **e**, **g**) Fluorescence intensity (FI) curves with penetration depth. (**b**, **d**, **f**, **h**) AUC of each group. Statistical comparisons were made using one-way ANOVA with Tukey’s multiple comparisons; ns no statistical significance, ^*^^*^p ≤ 0.01 and ^*^^*^^*^p ≤ 0.001. Data presented as the means ± standard deviation, *n* = 6. *R110* rhodamine 110, *R110@SNF* 0.03 wt% R110-laden silk nanofiber hydrogel, *HSs* hypertrophic scars, *AUC* area under the fluorescence intensity curves, *ANOVA* analysis of variance

### Statistical analysis

All error bars represent standard deviations. Data analyses were conducted using GraphPad Prism 9.0 software. When comparing more than two samples, either one- or two-way analysis of variance was used, with Tukey’s multiple comparisons test. *P* values <0.05 were considered statistically significant.

## Results

### Characterization of R6G@SNF and R110@SNF

AFM images showed the morphology changes of SNF after drug loading. Similar to the results of a previous study [31], the loaded R6G and R110 induced slight increases in SNF diameter without significant aggregation, indicating homogeneous drug distributions (Figure 1a, b, c). In addition to the typical peaks of SNF at ~1666 cm^−1^, the Raman spectrum of R6G@SNF exhibited the typical peaks of R6G at ~1367 and 1515 cm^−1^, while that of R110@SNF showed the typical peaks of R110 at ~1368 and 1506 cm^−1^ (Figure 1e, f). The results confirmed the successful loading of drugs on the SNFs. After the introduction of R6G and R110, the SNFs maintained their shear-thinning capacity, similar to drug-free SNF (Figure 1g). The physical appearance of SNF, R6G@SNF and R110@SNF are shown in Figure 1d.

### Cytocompatibility of SNF

Previous studies have revealed that SNF, which is derived from natural protein, had an antioxidant capacity and could attenuate the toxicity of the loaded drugs [31]. The effects of SNF on HDF and HKF cell viability were assessed by CCK-8 assays. When cultured for 1, 3, 5 and 7 days, both the HDF and HKF cells cultured with SNFs at different concentrations exhibited excellent viability (Figure 2a, b). These results suggested the excellent cytocompatibility of SNF. SNF as a natural compatible carrier is a preferred option for *in vivo* experiments.

### Penetration of R6G@SNF and R6G in rabbit ear HSs *in vivo*

The successful rabbit ear HS model is shown in Figure 3b. R6G as a hydrophilic drug model could be visualized directly through fluorescence. To reveal the penetration of the water-soluble drugs in the laser channels, the fluorescence intensity at the penetration depth in the peri-coagulation zone (peri-CZ) (the tissue around the CZ) and the intensity mean value in the CZ were measured separately (Figure 3a). As for the control groups without laser treatment, we only measured the fluorescence intensity at the penetration depth. We found that the red fluorescence was more concentrated in the epidermis and dermis for the laser-treated groups (Figure 4a), while the red fluorescence was concentrated only in the epidermis for the groups without laser irradiation (Figure 4b). According to the results of the fluorescence quantitative curve (Figure 5a, c, e, g) and the area under the curve (representing the total penetration of drugs in the peri-CZ, Figure 5b, d, f, h), L + R6G@SNF had more penetration through the laser channels compared to L + R6G on day 1 (Figure 5a, b; day 1 L + R6G@SNF: 5987 ± 900 vs day 1 L + R6G: 3941 ± 919.8, in 6 HSs, *p* < 0.0001). This indicated that R6G@SNF could significantly enhance the penetration of R6G with the beneficial application of a CO_2_ fractional laser within 24 h. However, the effect of SNF was less effective in the following days. For the groups without laser treatment, the dermis was almost completely impermeable to R6G@SNF and R6G, and no significant difference was found between the R6G@SNF and R6G groups (Figure 5). Additionally, for the laser treatment groups, the mean fluorescence intensity of the CZ was high and virtually unchanged on days 1, 3 and 5 (Figure 4b; all wounds lost their residual CZ on day 7), signifying a sustained high-drug-concentration in the CZ. Overall, with the aid of laser irradiation, R6G@SNF could effectively enhance transdermal delivery in the rabbit ear HS model.

### Penetration of R110@SNF and R110 in rabbit ear HSs *in vivo*

The analysis procedure was the same as the methods described before. Unlike R6G, R110 is insoluble in water but soluble in ethanol. We found that in the control groups, R110 was mainly concentrated in the stratum corneum (Figure 6a) and the R110 group had a higher concentration than the R110@SNF group on day 1 (Figure 7a, b; day 1 R110: 1629 ± 259.9 > day 1 R110@SNF: 1024 ± 226.2, *p* < 0.001). However, the results for these two groups on day 3 were reversed (Figure 7c, d; day 3 R110@SNF: 349.4 ± 72.80 > day 3 R110: 211.0 ± 55.57, *p* < 0.01). This indicated that the effects of the R110@SNF treatment may be sustained longer on intact skin. For the laser treatment groups, R110 was mainly concentrated in the CZ (Figure 6a) but had limited penetration in the peri-CZ regardless of whether SNF was used (Figure 7). Similar to R6G, there was plenty of R110 storage in the CZ, with no significant difference between each group (Figure 6b). This result illustrates that the CO_2_ fractional laser, to some extent, was beneficial for the direct entrance of R110@SNF and R110 into the skin.

## Discussion

This study characterized the distribution of SNFs loaded with R6G and R110 as hydrophilic and hydrophobic model drugs based on CO_2_ fractional laser treatment. Researchers divided the zone irradiated by the laser beam into two parts: one was the microscopic ablation zone (MAZ), which was the zone ablated by the laser showing an ‘empty’ zone of the laser channel, and the other was the CZ, a rim of thermally coagulated tissue of variable thickness surrounding the individual microchannels, which reflected residual thermal damage [33–35]. Many earlier studies did not separately consider the permeation of the CZ and peri-CZ, whereas we respectively analyzed the fluorescence intensities of the CZ and peri-CZ as the CZ was an inactivated tissue. The current study was conducted *in vivo* in a rabbit ear HS model for up to 7 days, with dynamic observation of drug delivery through confocal laser scanning microscopy. In particular, we provided a comprehensive comparison of the efficacies of CO_2_ fractional laser and SNF treatment regarding drug penetration in HSs. In addition, we studied the permeation of hydrophobic and hydrophilic drugs separately to provide a more comprehensive analysis of the permeation of both types of drugs in the laser channels and full-thickness skin. Collectively, our study concluded that SNF promoted the permeability of R6G in the laser channels within 1 day, but the same was not concluded for R110.

In our experiments, R6G@SNF with CO_2_ fractional laser treatment had the best penetration in the peri-CZ at day 1 compared to the other three groups. This result indicated that CO_2_ fractional laser irradiation was a key factor for drug penetration. CO_2_ fractional laser irradiation not only removes the epidermal barrier directly but also enhances the total area of absorption [35,36]. These features enable laser treatment to be more effective in topical drug penetration. The result also implied that SNF could promote R6G penetration. SNFs with a size of ~40 nm could penetrate across the corneum and be used as a transdermal carrier [37]. The prepared SNF here had a smaller size than the reported silk nanoparticles, suggesting its feasibility as a transdermal carrier [31]. A recent study conducted by our group found that water-soluble drugs loaded on SNF entered cells in two ways [38]. Some drugs were released slowly from the SNF and then permeated into the cells. The cells could also internalize the drug-laden SNF, allowing the other drugs still immobilized on the SNF to also enter the cells through this internalization. Since SNF could penetrate the skin and cell membranes, similar to what happened during cell culture, R6G probably entered the skin tissues in two ways when the skin was treated with laser irradiation. Considering that a single SNF could be loaded with a substantial amount of R6G and penetrate the tissues simultaneously, R6G showed deeper penetration when loaded on the SNF. In addition, the channels produced by laser irradiation reached the dermis with a high water content, into which water-friendly substances may penetrate better [35]. Likewise, several studies in related fields demonstrated that laser channels could provide a greatly enhanced uptake of topical 5-fluorouracil (5-FU), cisplatin and methotrexate [24,39,40], all of which are hydrophilic.

Meanwhile, for hydrophobic molecules such as R110, our study found that although R110@SNF and R110 solution could fill the channels, their penetration into the peri-CZ tissue was difficult. This illustrated that CO_2_ fractional laser irradiation, to some extent, was beneficial for the direct entrance of R110@SNF and R110 into the skin. Moreover, due to hydrophobic interactions, this penetration was difficult to enhance. Similarly, Olesen *et al.* found that the permeability of the highly lipophilic drug vismodegib is only modestly enhanced by CO_2_ fractional laser irradiation [41]. Additionally, for intact skin, R110@SNF appeared to have no effect on promoting R110 permeability compared to R110 ethanol on day 1. We speculated the R110 could adhere to SNF but could not be easily released because of these hydrophobic interactions. However, R110@SNF had a more sustained penetration on day 3 compared to R110, which may be due to R110@SNF adhering to the skin longer. Previous studies revealed that the size of nanocarriers is a critical factor in determining their transdermal capacity [27]. Improved penetration capacity in cancer cells and normal skin has been achieved for SNF when its size decreased from 1700 nm to <100 nm [38]. Although the SNF loaded with hydrophobic R110 showed inferior transdermal behavior, it could be possible to enhance the permeability of hydrophobic drugs by loading the drugs on SNFs with smaller sizes, which will be investigated in our future studies.

In previous investigations, the effect of CO_2_ fractional laser irradiation on increasing drug penetration began to fade after 24 h [26], which was mostly owing to the rapid re-epithelialization caused by the lateral migration of corneocytes [42]. Our findings were consistent with those of this prior study. With the epidermis and dermis gradually repaired, the MAZ closed and the CZs containing high drug concentrations migrated outside of the epidermis and were finally transformed into scabs. Wenande *et al*. argued that a CZ of an appropriate thickness could serve as a reservoir for a small hydrophilic drug, promoting drug diffusion into the surrounding skin [35]. Research about CZ thickness found that the uptake of topical compounds was higher through microchannels surrounded by a CZ than for those without a CZ. Moreover, the highest skin uptake of compounds was observed for a CZ of 20 μm, compared to that of CZs of 80 and 0 μm [43]. In our study, the CZ thickness was ~25 μm (data not shown), which was a suitable thickness for promoting penetration. It is noteworthy that the observation time window of these studies was limited to a few hours *in vitro.* However, in this *in vivo* study, we found that for a long time, drugs could not permeate through the CZ. We speculated that rapid re-epithelialization of the CZ may be the key to unsustainable drug release. Another potential reason might be that the function of vascular/lymphatic systems and metabolism is to contribute to drug elimination [35].

Recently, a randomized clinical trial found that no significant long-term difference in scar flattening was observed between a laser + corticosteroid group and the control group [44]. However, another clinical trial reached a different conclusion: that combining CO_2_ fractional laser irradiation and topical 5-FU or verapamil hydrochloride treatment resulted in a significant scar treatment effect compared to the control group [45]. We speculated the main reason for these different results was that 5-FU and verapamil hydrochloride are hydrophilic while corticosteroid is hydrophobic, which validates our results.

The treatment of abnormal scar tissue remains a great challenge in clinical medicine since few carriers can penetrate scar tissue to enhance the biological function of the loaded drugs. Developing a transdermal carrier that can be loaded with drugs with distinct properties and penetrate scar tissue is critical for improving the efficacy of abnormal scar treatment [26]. SNF could be loaded with and immobilize both hydrophobic and hydrophilic cargos and achieve controllable and sustained release [31]. Further research indicated that SNF could penetrate the corneum layer and cell membrane by tuning its size and shape, indicating its possible applications in scar treatment [37,38,46]. To verify the feasibility of SNF as a transdermal carrier in scar tissue, rhodamine, a biological dye, was loaded on SNF to visualize the SNF distribution in the scar tissue. Since the permeability of SNF in scar tissue has been proved, in a future study bioactive drugs will be loaded on carriers to induce scar changes and the results will be evaluated. In conclusion, we demonstrated a novel method of topical drug delivery. Our results suggested that CO_2_ fractional laser-assisted drug delivery is more suitable for the delivery of hydrophilic drugs. In addition, SNF could serve as a penetration enhancer for hydrophilic drugs.

## Conclusions

In summary, this study explored CO_2_ fractional laser-assisted drug-laden SNF delivery in a rabbit ear HS model. Our data indicated that the laser treatment group R6G@SNF exhibited better penetration in the peri-CZ compared to R6G on day 1, while R110@SNF exhibited less penetration in the peri-CZ. The mean fluorescence intensity of the CZ was dramatically high and virtually unchanged on days 1, 3 and 5 for the laser-treatment groups. For the groups without laser treatment, this fluorescence intensity was weak. We concluded that combining CO_2_ fractional laser irradiation with SNF drug delivery could improve the efficiency of R6G (hydrophilic drug) delivery within 1 day (before total re-epithelialization). In contrast, the penetration of R110 (hydrophobic drug) was limited in the peri-CZ. A major problem that hindered the long-time permeability of the drugs was rapid re-epithelialization. The major contribution of this study was combining CO_2_ fractional laser irradiation with SNF drug delivery to improve the efficiency of hydrophilic drug delivery, offering a novel idea for the topical administration of water-soluble drugs. To our knowledge, this is the first time that SNF and CO_2_ fractional laser irradiation have been combined to promote drug penetration.

## Abbreviations

AFLs: ablative fractional lasers; AFM: atomic force microscopy; CCK-8: cell counting kit 8; CLSM: confocal laser scanning microscopy; CZ: coagulation zone; 5-FU: 5-fluorouracil; HDF: human dermal fibroblasts; HKF: human keloid fibroblasts; HS: hypertrophic scar; MAZ: microscopic ablation zone; peri-CZ: peri-coagulation zone; R110: rhodamine 110; R6G: rhodamine 6G; SNF: silk nanofiber hydrogel.

## Data Availability

The dataset(s) supporting the conclusions of this article may be obtained from the corresponding author.
